# Supplementary material for: Development of mental health first aid guidelines for psychosis for Brazil: A Delphi expert consensus study
Source: PLoS One. 2024 Jul 22;19(7):e0307064. doi: 10.1371/journal.pone.0307064 (PMC11262670; doi:10.1371/journal.pone.0307064)
Supplement: S2 File — Final guidelines document in Brazilian-Portuguese language. (PDF) [file pone.0307064.s002.pdf]

O QUE É A PSICOSE?

Psicose é um termo amplo para descrever um problema de saúde mental em que uma pessoa perde o contato com a realidade. Existem graves distúrbios nas emoções, pensamentos e comportamentos. A psicose pode ter um impacto grande na vida das pessoas, assim como em seus relacionamentos, trabalho ou estudos. A psicose pode ser muito estressante e perturbadora para a pessoa e para aqueles que convivem com ela. É importante saber que o tratamento é mais eficaz quando a psicose é detectada precocemente.

Os sintomas da psicose podem se apresentar como um episódio único ou como parte do percurso de uma doença, pois podem estar presentes em diversos transtornos mentais, como esquizofrenia, transtorno bipolar ou demência. Os sintomas da psicose também podem estar atrelados ao uso de álcool ou drogas. É importante saber que as pessoas que apresentam psicose ou esquizofrenia não têm ‘dupla personalidade’, nem se trata de uma deficiência intelectual, e muito menos, de algo contagioso.

Existem vários fatores que podem agir como gatilhos para a psicose, como o uso de substâncias, estresse extremo ou traumas. É possível que a pessoa não saiba, não perceba ou não aceite o que está acontecendo com ela ou que não está bem. Saiba que a pessoa está vivenciando sintomas que estão além do seu controle; no entanto, não a culpe nem leve suas ações para o lado pessoal.

É importante saber que a pessoa que está apresentando sinais ou sintomas psicóticos não tem necessariamente um transtorno psicótico. Tente aprender mais sobre a psicose através da busca de informações confiáveis, como em redes online reputáveis ou em organizações de saúde mental.

Essas diretrizes são para fornecer primeiros socorros de saúde mental a uma pessoa que está vivenciando sintomas psicóticos. As informações nestas diretrizes não envolvem situações de crise, exceto quando indicado ao contrário.

COMO SABER QUE ALGUÉM PODE ESTAR DESENVOLVENDO PSICOSE?

É importante estar apto a reconhecer os sinais e sintomas precoces da psicose, como mudanças abruptas no jeito de ser da pessoa. Um grupo de sinais e sintomas está mais associado a um transtorno psicótico do que um único sinal ou sintoma isoladamente. Os sinais e sintomas podem aparecer de repente ou desenvolver-se gradualmente ao longo do tempo, podendo variar de pessoa para pessoa e mudar de acordo com o período. A pessoa pode vivenciar múltiplos episódios de psicose com períodos de bem-estar entre eles.

Se você não tiver certeza se a pessoa está tendo um episódio psicótico, busque ajuda de um(a) profissional de saúde mental ou pergunte aos amigos e familiares da pessoa se já houve algum episódio anterior ou diagnóstico de psicose. Questione amigos e familiares sobre a percepção de alguma mudança de comportamento preocupante em relação à pessoa.

SINAIS E SINTOMAS COMUNS QUANDO A PSICOSE ESTÁ SE MANIFESTANDO[1]

Alterações na emoção e motivação:
Depressão; ansiedade; irritabilidade; desconfiança; emoção embotada, plana ou inadequada; mudança no apetite; energia e motivação reduzidas.

Mudanças no pensamento e percepção:
Dificuldades de concentração ou atenção; sensação de alteração de si mesmo, dos outros ou do mundo exterior (por exemplo, sentir-se diferente ou sentir que os outros mudaram ou estão agindo de forma diferente); ideias estranhas; experiências perceptivas incomuns (como, por exemplo, redução ou aumento de cheiro, som ou cor).

Mudanças de comportamento:
Distúrbios de sono; isolamento ou afastamento social; capacidade reduzida para desempenhar papéis sociais ou de trabalho.

[1] Adaptado de*: Edwards, J & McGorry, PD (2002). Implementing Early Intervention in Psychosis. Martin Dunitz, London*.

PONTOS A SEREM EVITADOS CASO VOCÊ ACREDITE QUE UMA PESSOA ESTÁ VIVENDO COM PSICOSE:

- Não ignore ou dispense sinais e sintomas, mesmo se aparecerem gradualmente ou não forem claros. Por exemplo, a falta de motivação da pessoa pode ser um sintoma de psicose, ao invés de preguiça.
- Não assuma que uma pessoa que apresenta sinais e sintomas psicóticos está "apenas passando por uma fase", vivenciando os altos e baixos da vida ou apenas usando drogas de forma recreacional.
- Não ache que os sinais e sintomas irão se dissipar sozinhos.

COMO DEVO ABORDAR A ALGUÉM QUE POSSA ESTAR VIVENCIANDO PSICOSE?

A pessoa que está desenvolvendo psicose pode não buscar por ajuda. Preste atenção aos sinais de comunicação não-verbal da pessoa. Se você não conhecer a pessoa muito bem, não deixe que isto atrapalhe a sua abordagem com ela. O importante é que isto ocorra num ambiente seguro, confortável e livre de distrações. Reserve um tempo adequado para conversar com a pessoa para não ser uma conversa apressada. Tente estar calmo(a), independente do seu estado emocional pessoal. Tente achar um momento que ambos (a pessoa e você) estejam calmos. Aborde a pessoa de modo gentil e sem julgamentos, de uma forma não confrontadora. Tente adaptar a sua abordagem e interação conforme o comportamento da pessoa, como por exemplo, se a pessoa estiver desconfiada e evitando contato direto com o seu olhar. Esteja atento a isto e dê o espaço que a pessoa precisa.

COMO DEVO CONVERSAR COM A PESSOA SOBRE OS SEUS SINTOMAS?

Primeiramente, pergunte à pessoa se ela quer conversar sobre como ela está se sentindo. Caso sim, tente encontrar uma base comum para a discussão, gradualmente construindo perguntas mais específicas sobre o que a pessoa está vivenciando, sempre demonstrando empatia durante a conversa. Você pode perguntar à pessoa por quanto tempo ela tem percebido estes sintomas, ou, de uma forma gentil, sobre aquilo que tem afetado o comportamento dela (como por exemplo, ouvir vozes ou ter pensamentos estranhos/não usuais).

É possível que a pessoa seja vaga em relação aos seus sintomas, dando mais ênfase aos sintomas físicos em detrimento dos sintomas mentais. Caso você tenha notado mudanças no comportamento da pessoa, pergunte a ela se isso a incomoda. Permita que a pessoa fale sobre suas experiências, sentimentos e crenças, se ela assim desejar.

Não use o termo ‘psicose’, mas sim discuta suas preocupações sobre pensamentos, sentimentos ou comportamentos que notou na pessoa. Não seja agressivo fisicamente ou verbalmente com a pessoa. Seja o mais amigável possível. Se você souber que a pessoa usa algum medicamento prescrito para psicose, pergunte se ela ainda está tomando o medicamento. Caso ela diga que não está tomando a medicação, encoraje-a a tomar.

DURANTE A CONVERSA COM A PESSOA SOBRE O QUE ELA ESTÁ VIVENCIANDO, SIGA AS DICAS DE BOA COMUNICAÇÃO ABAIXO:

DICAS PARA CONVERSAR COM ALGUÉM QUE PODE ESTAR VIVENCIANDO PSICOSE:
**Linguagem verbal**<br>• Use perguntas simples e direta.<br>• <u>Não</u> use termos estigmatizantes que possam levar a pessoa à defensiva, como louco(a) ou psicótico(a).<br>• <u>Não</u> discuta crenças sobre religião ou saúde mental.<br>**Ouvindo sem julgamento**<br>• Ouça sem julgamentos.<br>• Ouça atentamente e reflita acerca do que ouve, demonstrando através do uso de palavras como “sim” e “entendo”.<br>• Repita o que a pessoa disse usando outras palavras, como forma de checar que está entendendo corretamente o que foi dito.<br>• Faça perguntas de esclarecimento, assim mostrando que está ouvindo.<br>**Linguagem corporal**<br>• Não toque a pessoa sem a sua permissão.<br>• Mantenha contato visual durante a conversa, mas sem ficar encarando a pessoa.<br>• Minimize gestos de desconforto ou nervosismo, como inquietação, balançar as pernas ou roer as unhas.<br>• Se a pessoa estiver sentada, não fique em pé ou rondando em torno da pessoa.

COMO POSSO SER SOLIDÁRIO(A) E COMPREENSIVO(A)?

Pergunte à pessoa se, e como, ela gostaria que você a ajudasse. Assegure à pessoa que você está lá para dar o suporte necessário, ajudando e mantendo-a segura. Deixe claro para a pessoa que você está disposto e apto a ajudá-la.

Apoio/suporte social pode ser de grande ajuda para a pessoa; contudo, ela pode ter um déficit no suporte social porque ela tende a se isolar ou seu comportamento acaba afastando as pessoas dela.

Se for apropriado ao relacionamento, pergunte à pessoa se está tudo bem entrar em contato com ela de tempos em tempos. Se você tiver contato contínuo com a pessoa, observe sinais que possam indicar piora nos sintomas. Contudo, durantes estas interações, não foque somente nos problemas de saúde mental da pessoa. Não recorra a ameaças com o objetivo de mudar o comportamento da pessoa.

Se a pessoa estiver muito insegura, faça companhia à ela, assegurando de que ela não está sozinha. Caso a pessoa esteja angustiada por seus sintomas, tente confortá-la. Evite ser negativo ou pessimista quando estiver falando com a pessoa em relação ao seu futuro.

Se a pessoa for um(a) adolescente, fique com ele(a) enquanto ele(a) entra em contato ou fala com um parente ou adulto de confiança.

COMO POSSO TRATAR A PESSOA COM DIGNIDADE E RESPEITO?

É importante sempre tratar a pessoa com respeito. Reconheça a coragem da pessoa em conversar com você. Evite usar termos triviais quando estiver interagindo com a pessoa, como por exemplo, “levante-se”, “eu tenho certeza que isso vai passar” ou “poderia ser pior”. Não repudie ou ridicularize a pessoa, mesmo que o que ela esteja dizendo não faça sentido para você. Seja tolerante com as mudanças no comportamento da pessoa, ao menos que seu comportamento se torne perigoso ou inapropriado. Não demonstre raiva ou frustração caso você venha a sentir em relação à pessoa. Se a pessoa chatear-se com algo que você disse ou fez, peça desculpas e reconheça os sentimentos da pessoa.

Se outras pessoas estiverem presentes enquanto você conversa com a pessoa, não fale sobre a pessoa como se ela não estivesse ali. Contudo, é importante respeitar a privacidade e direito à confidencialidade da pessoa, a menos que a pessoa esteja em risco para si ou outrem.

COMO LIDAR COM SITUAÇÕES DE ALUCINAÇÕES E DELÍRIOS?

O QUE SÃO ALUCINAÇÕES E DELÍRIOS?

Alucinações são percepções falsas e normalmente envolvem ouvir vozes, mas também podem envolver (falsamente) enxergar, sentir, gosto ou cheiro de coisas.

Delírios são falsas crenças, como de perseguição, ter uma missão especial ou estar sob controle externo.

Adaptado de: *Mental Health First Aid Australia. Psychosis: first aid guidelines (revised 2019).Melbourne: Mental Health First Aid Australia; 2019*

É importante saber que delírios e alucinações parecem reais para a pessoa. Pergunte a pessoa se ela quer falar sobre o que está vendo ou ouvindo. Caso ela queira falar sobre suas alucinações ou delírios, é importante ouví-la de uma forma que mostre empatia, desenvolvendo um entendimento em relação ao que ela está vivenciando. Incentive a pessoa a considerar a evidência dos seus delírios através do questionamento sem críticas e nem julgamentos.

Se a pessoa estiver ouvindo vozes, ela pode reagir através de comportamentos como falar com si mesma, sussurrar para si mesma, em resposta às vozes que está ouvindo. Se existirem aspectos do ambiente em volta da pessoa que pareçam aumentar as suas alucinações ou delírios, limite ou remova esses aspectos quando possível. Até que você saiba o conteúdo dos delírios da pessoa, é importante que você se mantenha seguro(a) de possíveis reações agressivas. Se você observar alguma pessoa fazendo piadas ou criticando a pessoa, peça que parem. É importante ter empatia em relação a como a pessoa se sente, às suas crenças e experiências, sem estabelecer julgamentos sobre o conteúdo dessas crenças e experiências.

E SE A PESSOA ESTIVER NUM MOMENTO DE PARANÓIA?

As experiências da pessoa com alucinações ou delírios podem levá-las a não confiar nas pessoas, mesmo nas que são próximas dela. Se a pessoa estiver num momento de paranóia, você deve:

- Dê instruções simples à pessoa, se necessário, como por exemplo: "sente-se e vamos conversar sobre isso".
- Diga à pessoa o que você vai fazer antes de fazê-lo, como por exemplo que você vai pegar o telephone agora.
- Fique com a pessoa, mas numa distância que seja agradável tanto para a pessoa quanto a você.

Se a pessoa estiver paranoica, não dencoraje ou acalore o que a pessoa está vivendo, como por exemplo, sussurrando para ou sobre a pessoa.

SE A COMUNICAÇÃO DA PESSOA ESTIVER AFETADA?

A pessoa experienciando psicose pode não ser capaz de se comunicar da forma como ela faria normalmente. Por exemplo, a pessoa pode responder perguntas não relacionadas, pular de um assunto para outro ou estar ouvindo vozes, dificultando a sua comunicação. A pessoa pode não perceber linguagem não-verbais, como a expressão facial ou tonalidade da voz. No entanto, evite demonstrações excessivas de emoções (tanto positivas quanto negativas) ou o uso de linguagem complexa (como metáforas e sarcasmo).
Tente se comunicar claramente e de forma simples, repetindo o que foi dito, se necessário. Dê tempo suficiente para a pessoa responder perguntas, caso ela tenha dificuldade em processar a informação.
Se a pessoa estiver mostrando uma limitação de sentimentos, saiba que isto não significa que isso é tudo o que a pessoa está sentindo.

COMO LIDAR COM PROBLEMAS DURANTE A CONVERSA?

Mesmo que a pessoa perceba que ela não está bem, a confusão e o medo sobre o que está acontecendo pode levar a pessoa a negar que há algo de errado com ela. Saiba que a pessoa pode estar assustada com seus pensamentos e sentimentos. É possível que diversas conversas sejam necessárias antes da pessoa se abrir com você.

Se você achar irritante o comportamento da pessoa, entenda que a situação pode ser mutuamente angustiante. Se a conversa com a pessoa se tornar estressante ou emocionalmente desafiante, faça uma pausa para que você e a pessoa se acalmem.

Se a pessoa estiver angustiada por suas experiências, pergunte a pessoa o que iria ajudá-la a se sentir segura e no controle da situação. É possível que a pessoa reaja com emoções que não parecem adequadas ao contexto da conversa, como por exemplo, dar risadas.

Se a pessoa negar que tem algo de errado ou não quiser falar sobre o que está acontecendo com ela, você deve:

- Concentrar-se em ouví-la ao invés de tentar mudar o que ela pensa.
- Perguntar à pessoa se tem alguma coisa que você pode fazer para ajudá-la.
- Falar pra pessoa que ele(a) estará disponível para conversar no futuro.
- Apontar mudanças que você notou no comportamento dela.

Se a pessoa negar que tem algo de errado ou não quiser falar sobre o que está acontecendo com ela, **não discuta** com a pessoa.

AJUDA PROFISSIONAL

Saiba quais serviços estão disponíveis localmente e tenha um conhecimento geral/amplo sobre os tipos de tratamento que podem ser úteis para a psicose. Saiba o método de encaminhamento para ajuda profissional, como encaminhamento de um médico generalista ou médico de família para um especialista (quando usando o sistema público de saúde).

COMO POSSO INCENTIVAR A PESSOA A BUSCAR AJUDA PROFISSIONAL?

Sujira que a pessoa procure ajuda profissional, dizendo que:

- O que ela está vivenciando pode melhorar com ajuda profissional apropriada.
- Procurar ajuda profissional assim que possível é importante para impedir que os sintomas dela piorem
- Procurar ajuda profissional não significa que a pessoa será necessariamente hospitalizada, visto que o tratamento precoce pode ser realizado fora do hospital (na comunidade).
- Os profissionais da saúde estão do lado dela.
- É algo bom buscar ajuda, isto é um sinal de força e não de fraqueza ou falha.

Foque em sintomas específicos que estão preocupando a pessoa, enfatizando como o tratamento pode ajudá-la. Se a pessoa não confiar no profissional que a ajudou primeiro, incentive ela a buscar uma segunda opinião de um outro profissional. Saiba que embora você tenha o direito de expressar suas preocupações sobre a pessoa para um profissional de saúde e solicitar assistência, o profissional deve manter a confidencialidade, portanto é improvável que este compartilhe qualquer informação ao caso.

Deixe claro que o médico generalista/de família da pessoa pode ser um bom ponto de partida na busca de ajuda profissional. Pergunte à pessoa se ela possui um(a) médico(a) de confiança, e caso sim, encoraje a pessoa a buscar ajuda com o(a) mesmo(a). Diga a pessoa que, se ela procurar ajuda profissional com um médico generalista ou médico de família, ela vai receber um encaminhamento e pode ser referenciada a um serviço especializado. Encoraje a pessoa a ver seu(sua) médico(a) para um check-up, pois sintomas psicóticos podem se originar de doenças orgânicas.

Não ameaçe, confronte ou pressione a pessoa quando encorajando ela a buscar ajuda profissional. Esteja ciente da influência que a família da pessoa pode ter, por exemplo, a família pode encorajar ou desencorajar a pessoa a obter o tratamento que ela precisa.

Se a pessoa for um(a) adolescente, assegure-se de que o(a) adolescente consiga uma consulta com um profissional de saúde.

COMO POSSO APOIAR A PESSOA A BUSCAR AJUDA PROFISSIONAL?

Se for apropriado para a relação, assegure a pessoa que você irá apoiá-la enquanto ela buscar e receber ajuda profissional. Caso a pessoa possua uma consulta, pergunte se ela gostaria que você, alguma outra pessoa ou amigo acompanhasse ela na consulta.

Continue incentivando a pessoa a buscar ajuda profissional, mesmo que surjam desafios na obtenção do cuidado ou que você não tenha certeza de que a pessoa esteja vivenciando psicose. Se a pessoa estiver tendo dificuldade em conseguir conselhos ou ajuda, incentive a pessoa a contatar uma agência de suporte em saúde mental como um CAPS (Centro de Assistência Psicossocial).

E SE A PESSOA NÃO QUISER AJUDA PROFISSIONAL?

Se a pessoa não quiser procurar ajuda profissional, mantenha-se paciente, pois as pessoas que vivenciam psicose geralmente precisam de tempo para reconhecer que não estão bem. É possível que a pessoa não queira buscar ajuda profissional porque ela acredita que as pessoas estão tentando machucá-las. Expresse calmamente sua preocupação em relação à escolha da pessoa de não procurar ajuda, e as possíveis implicações em relação à isto, enfatizando os benefícios de obter ajuda (por ex: melhora na ansiedade ou nos sintomas que a assustam). Encoraje a pessoa a falar com alguém de confiança sobre o que ela está vivenciando.
Se a pessoa não reconhecer que ela não está bem, ela pode ativamente resistir às suas tentativas de encorajá-la a procurar ajuda. No entanto, você deve:

- Buscar informações com outras pessoas que tenham experiência em incentivar pessoas com sintomas psicóticos a buscar ajuda;
- Consultar “experts” para saber aconselhar e dar o melhor suporte possível para a pessoa em busca de ajuda profissional;
- Falar com a família ou amigos próximos da pessoa, de modo que eles sejam capazes de facilitar os próximos passos para obtenção de ajuda profissional para a pessoa.

A pessoa tem o direito de recusar tratamento, a menos que ela possua critério para tratamento involuntário. Contudo, nunca ameaçe a pessoa com tratamento involuntário ou hospitalização. Informe a pessoa opções de ajuda em sua comunidade, como visitas domiciliares ou serviços comunitários. É importante manter uma boa relação com a pessoa, de forma que ela possa procurar a sua ajuda no futuro.

Se você discutir as suas preocupações em relação a pessoa com um profissional de saúde, descreva claramente suas observações (por ex: diga exatamente o que a pessoa estava fazendo e falando, onde e quando) de modo que o(a) profissional tenha acesso a todas as informações necessárias. Caso você venha a compartilhar informações sobre a pessoa com um(a) profissional de saúde, peça que o profissional mantenha confidencialidade/sigilo e use estas informações de forma coerente e sensível, protegendo a sua relação com a pessoa.

Mantenha uma lista de telefones de emergência, como de um UPA (Unidade de Pronto Atendimento 24h) e também telefones de emergência psiquiátrica.

E AUTO-AJUDA E OUTROS TIPOS DE APOIO?

Pergunte a pessoa se ela já se sentiu dessa maneira antes e se a resposta for afirmativa, pergunte o que foi útil para a sua melhora. Encoraje a pessoa a se engajar em um modo de vida saudável, como exercícios físicos regulares, dieta saudável e abandonar o uso de substâncias. Caso seja apropriado ao relacionamento, encoraje a pessoa a cuidar de sua saúde física, como por exemplo, mantendo um estilo de vida saudável e fazendo check-ups médicos regulares.
Informe a pessoa que existem programas que provém suporte educacional e para busca de empregos, se isso for importante a ela.
Tente perceber se a pessoa tem um bom suporte social e se a resposta for afirmativa, encorajá-la a usar esse suporte.

E SE FOR UMA MÃE QUE DEU À LUZ RECENTEMENTE?

A psicose pós-natal é uma condição na qual os sintomas da psicose começam de repente nas primeiras semanas após o parto.

Psicose pós-natal pode ter uma rápida progressão e, sem tratamento imediato, pode levar a riscos tanto para a mãe quanto para o bebê. Se você acha que uma mãe pode estar vivenciando psicose pós-natal, ou tendo delírios que envolvam seu bebê, ligue para um serviço especializado em saúde mental imediatamente. Tente envolver o(a) parceiro(a) da mãe ou a família dela de modo a minimizar o risco para ela e para o bebê. Assegure-se de que a mãe esteja sempre acompanhada até receber a ajuda profissional de que necessita.

E SE A PESSOA ESTIVER USANDO ÁLCOOL OU OUTRAS DROGAS?

Desencoraje a pessoa a usar álcool ou outras drogas, e diga a ela que álcool ou outras drogas podem fazer seus sintomas piorarem. Se você acredita que a pessoa está com problemas de uso de álcool, você deve seguir as *Diretrizes de Primeiros Socorros de Saúde Mental Para Ajudar Alguém Com Problemas de Uso de Álcool no Brasil.* Para baixar as diretrizes, clique em "Additional file 2" dentro do link <Diretrizes de Primeiros Socorros de Saúde Mental Para Ajudar Alguém Com Problemas de Uso de Álcool no Brasil>.

E SE A PESSOA ESTIVER NUM ESTADO PSICÓTICO GRAVE?

Uma pessoa está num estado psicótico grave se ela estiver tendo delírios e alucinações perturbadores, pensamentos muito desorganizados ou comportamentos bizarros e perturbadores. Elas podem parecer muito angustiadas, seus comportamentos podem ser perturbadores para outras pessoas ou podem se comportar de uma maneira que põe em perigo a si mesmos ou a outros. Elas podem ou não se comportar de forma agressiva. O comportamento agressivo pode variar desde abuso verbal a abuso físico e pode causar danos físicos ou emocionais a outras pessoas.[1]

Fonte: [1]*Mental Health First Aid Australia. Psychosis: first aid guidelines (revised 2019).Melbourne: Mental Health First Aid Australia; 2019*

CONSIDERAÇÕES DE SEGURANÇA QUANDO A PESSOA ESTÁ NUM ESTADO PSICÓTICO GRAVE

Se a pessoa estiver num estado psicótico grave ou se comportando de forma agressiva, aborde a pessoa com precaução. Você deve tentar proteger a pessoa, a si mesmo(a) e aos outros ao redor de você. Não tente argumentar com uma pessoa que está com quadro psicótico grave.

Esteja ciente de que a pessoa pode agir com base em uma alucinação ou delírio. Sua meta primária é manter a si e aos outros em segurança. Fique a uma distância segura da pessoa enquanto consegue manter a interação e assegurando-se que tem livre acesso a uma saída. É importante você levar a sério qualquer ameaça ou sinais, especialmente se a pessoa acreditar que está sendo perseguida. Se você estiver com medo, procure ajuda externa imediatamente, nunca se colocando em risco.

Se for seguro, tente limitar o acesso a meios que a pessoa possa usar para prejudicar a si mesmo e aos outros, removendo quaisquer armas ou objetos que possam ser usados como armas do ambiente em torno da pessoa. Se a pessoa tiver uma arma ou algo que possa ser usado como uma arma, não aborde a pessoa e ligue para os serviços de emergência imediatamente.

Se você estiver sozinho(a) com a pessoa, ligue pra outra pessoa para ficar com você até que uma ajuda profissional chegue. Se você estiver sozinho com a pessoa e não puder ficar mais com ela, chame alguém para ficar com ela até que uma ajuda profissional chegue.

DIMINUINDO A TENSÃO QUANDO A PESSOA ESTÁ NUM ESTADO PSICÓTICO GRAVE

Ouça mais a pessoa e fale menos. Permita que a pessoa expresse seus sentimentos e empatize com estes.

Fale com a pessoa usando frases simples e curtas. Fale calmamente, não eleve sua voz e nem grite com a pessoa. Mantenha-se calmo(a) e não demonstre medo ou ansiedade. Evite usar o celular enquanto estiver ajudando a pessoa.
Não faça nada que possa agitar a pessoa. Evite comportamentos ansiosos/nervosos, como ficar movendo/batendo os pés, mãos/dedos, ou movimentar-se bruscamente, falar rápido, etc. Se for necessário mover-se para perto ou realizar contato físico com a pessoa, peça permissão à pessoa, como: “Você se importa se eu sentar próximo a você?” ou “posso ver que seus braços estão machucados, tudo bem se eu usar o kit de primeiros socorros para colocar um curativo?”.

Se existir mais de uma pessoa presente, crie um ambiente agradável em torno da pessoa, de modo que ela não se sinta sufocada. Encoraje apenas uma pessoa a falar por vez. Se existir pessoas presentes que não exercem um papel ajudando no controle da crise, solicite que elas deixem o ambiente. Se a pessoa tiver medos irreais quanto a sua segurança, tranquilize ela afirmando que vocês estão seguros.

Tente obter informação para saber se a pessoa se sente segura, como: “Você parece preocupado, existe algo que eu possa fazer para te ajudar?” ou “Você se sente segura? Ou existe algo do qual você tem medo?”. Não leve nada que a pessoa disser para o pessoal. Diga a pessoa saber que você está ali para ajudar. Pergunte a pessoa no que você pode ajudar e tente encontrar o que faria a pessoa sentir-se mais segura e em controle. Se a pessoa tiver um plano de prevenção ou recaída, você deve segui-lo. Tente descobrir se a pessoa tem alguém em quem ela confia (por exemplo, amigos próximos ou família), caso sim, tente recrutar a ajuda deles.
Se a pessoa estiver consumindo álcool ou outras drogas, desencoraje a pessoa de consumir mais.

Se você não conseguir diminuir a tensão da situação, busque ajuda profissional, como por exemplo, serviços especializados em crises psiquiátricas ou serviços de emergência.

BUSCANDO AJUDA QUANDO A PESSOA ESTÁ NUM ESTADO PSICÓTICO GRAVE

Tente ter certeza de que a pessoa será avaliada por um profissional de saúde imediatamente. Explique para a pessoa porque você acredita que uma avaliação médica é necessária. Providencie opções em relação a busca de ajuda, pois isso pode dar a pessoa a sensação de controle, como “Você quer ir ao hospital comigo ou prefere que João te leve?” Se a pessoa já estiver recebendo ajuda profissional para transtorno psicótico, ligue para o profissional de saúde responsável imediatamente.

Se você ligar para um serviço de saúde mental, não rotule a pessoa como ‘psicótica’, ao invés disso, descreva os sintomas ou suas preocupações imediatas.

Se as suas preocupações em relação a pessoa forem rejeitadas pelos serviços que você contactou, tente novamente buscar apoio para a pessoa, como ligar pra outro serviço.

Se você suspeitar que a pessoa pode ser uma ameaça para si mesma ou para outros, ligue para o serviço de emergência imediatamente. Se você acha que a pessoa está em risco de suicídio ou em risco de machucar a si mesma, diga isso ao serviço de emergência.

Se você precisar ligar para o serviço de emergência, explique que a pessoa está precisando urgentemente de ajuda médica. Se você ligar para o serviço de emergência, explique que você está preocupado com o fato da pessoa estar passando por uma crise psicótica. Descreva de forma específica e concisa as observações em relação ao comportamento e sintomas da pessoa. Se a pessoa tiver sido diagnosticada anteriormente com um transtorno psicótico, explique isto ao serviço de emergência. Caso a pessoa estiver armada ou se houver armas acessíveis por perto, informe o serviço de emergência.

Se o serviço de emergência responder/atender, tente encontrá-los assim que chegarem, para que você possa explicar a situação para eles antes deles se aproximarem da pessoa. Se a polícia ou o pessoal do serviço de emergência chegar, esteja disponível para explicar a situação da pessoa.

Se a pessoa estiver num estado psicótico grave e recusar a procurar ajuda profissional, saiba que a pessoa mais próxima da pessoa pode solicitar avaliação de saúde mental. **Se a pessoa estiver em estado psicótico grave e não assumir que não está bem,** ligue ao serviço de emergência e peça por avaliação sob a legislação de saúde mental relevante.

E SE A PESSOA ESTIVER EM ESTADO PSICÓTICO GRAVE E PRECISAR IR AO HOSPITAL?

Se você acha que a pessoa precisa ir ao hospital, mas você não se sente seguro(a) para levá-la ao hospital, ligue pro serviço de emergência. Se a pessoa ameaçar a ferir a si mesma ou aos outros, não tente levá-la ao hospital sem a ajuda de uma outra pessoa.
Se a pessoa for ao hospital, e se for apropriado para a relação, tente falar diretamente com o(a) medico(a) ou equipe de emergência para prover informações relevantes a situação da pessoa. Se a pessoa precisar ser admitida ao hospital, apoie ela focando a conversa nos benefícios que o hospital poderá trazer, como diminuindo os seus dos sintomas.
Se a admissão ao hospital for recomendada por um profissional de saúde mental e a pessoa não concordar com isso, verifique se parentes ou amigos da pessoa conseguem persuadi-la. Caso você faça parte da família da pessoa, esteja preparado(a) para procurar tratamento involuntário (se for necessário). Informe-se das leis em relação ao tratamento involuntário.

E SE A PESSOA ESTIVER SE COMPORTANDO DE FORMA AGRESSIVA?

Pessoas em estados psicóticos não são usualmente agressivas e o risco de machucar a si mesma é muito maior do que machucar os outros. Portanto, não deixe a pessoa sozinha. Certos sintomas psicóticos como alucinações visuais e auditivas podem fazer com que a pessoa se torne agressiva.

Não ameace a pessoa, pois isso pode aumentar seu medo ou provocar um comportamento agressivo. Não responda de forma hostil, disciplinar, argumentativa ou desafiadora. Evite fazer muitas perguntas à pessoa, pois isso pode desencadear uma atitude defensiva e mais raiva ainda. Saiba que a agressividade da pessoa pode ser exacerbada por certas medidas que você tome, por exemplo, envolver a polícia.

Se a agressividade da pessoa estiver fora de controle, retire-se da situação e ligue para o serviço de emergência. Informe a polícia caso a pessoa possua uma arma.

E SE EU ACHAR QUE A PESSOA ESTÁ EM RISCO DE SUICÍDIO?

Se você achar que a pessoa está em risco de suicídio, siga as *Diretrizes Para Pensamentos e Comportamentos Suicidas no Brasil*. Para baixar as diretrizes, clique em "Additional file 2" dentro do link <Diretrizes Para Pensamentos e Comportamentos Suicidas no Brasil>.

Avalie o risco de danos à pessoa e aos outros. Se você achar que a pessoa **não está** em risco imediato, mas ainda assim estiver preocupado(a) com o seu bem-estar, pergunte a ela se existe alguém próximo que poderia ajuda-la a manter-se segura.

COMO EU POSSO ME CUIDAR?

É possível que você sinta uma gama de emoções (por ex: choque, confusão ou culpa) quando você perceber que alguém próximo a você está com sintomas de psicose, saiba que isso é comum. Também é comum você ter sentimentos negativos (por ex: choque, medo, tristeza, raiva e frustração) por ajudar alguém que está vivenciando psicose.
Você deve cuidar da sua própria saúde mental e bem-estar. Não se pressione nem pressione aos outros para achar soluções para todos os problemas da pessoa. Se você estiver achando o seu papel estressante, procure apoio através de grupos de suporte e organizações, profissional de saúde mental ou um amigo de confiança, mantendo confidencialidade sobre a pessoa. Tente estratégias de auto-ajuda para reduzir o stress, por ex: métodos de relaxamento, fazer exercício regularmente, dormir bem, e manter uma dieta saudável.

OBJETIVO DESTAS DIRETRIZES

Estas diretrizes foram elaboradas para ajudar o público a prestar primeiros socorros a alguém que possa estar sofrendo de psicose. O papel do socorrista é ajudar a pessoa até que ela receba ajuda profissional ou a sua crise se resolva.

Desenvolvimento destas Diretrizes

Estas diretrizes são baseadas no estudo conduzido com opiniões de pessoas com experiência vivida em psicose (consumidores e cuidadores) e profissionais de saúde mental (clínicos, pesquisadores e educadores) do Brasil. O estudo e diretrizes foram realizados através da colaboração da Mental Health First Aid (Austrália), Universidade de Melbourne (Austrália) e Universidade de São Paulo (Brasil).

A metologia usada no desenvolvimento destas diretrizes foi baseada no seguinte estudo: *Cottrill FA, Bond KS, Blee FL, Kelly CM, Kitchener BA, Jorm AF, Reavley NJ: Offering mental health first aid to a person experiencing psychosis: a Delphi study to redevelop the guidelines published in 2008. BMC psychology 2021, 9(1):1-15.*

Como usar estas Diretrizes

Essas diretrizes são um conjunto geral de recomendações. Cada indivíduo é único e é importante adaptar o seu apoio às necessidades da pessoa. Portanto, essas recomendações podem não ser apropriadas para todas as pessoas.

Embora essas diretrizes sejam protegidas por direitos autorais, elas podem ser reproduzidas livremente sem fins lucrativos, desde que a fonte seja citada.

Por favor, cite estas diretrizes da seguinte forma:

Mental Health First Aid Australia. Psicose: Diretrizes Primeiros Socorros (Brasil). Mental Health First Aid Australia; 2022.

Perguntas devem ser enviadas para: Mental Health First Aid Australia através do e-mail: mhfa@mhfa.com.au
